# Quantifying Adhesion Mechanisms and Dynamics of Human Hematopoietic Stem and Progenitor Cells

**DOI:** 10.1038/srep09370

**Published:** 2015-03-31

**Authors:** Alexandra S. Burk, Cornelia Monzel, Hiroshi Y. Yoshikawa, Patrick Wuchter, Rainer Saffrich, Volker Eckstein, Motomu Tanaka, Anthony D. Ho

**Affiliations:** 1 Physical Chemistry of Biosystems, Institute of Physical Chemistry, University of Heidelberg, 69120 Heidelberg, Germany; 2 Institute of Toxicology and Genetics, Karlsruhe Institute of Technology, 76021 Karlsruhe, Germany; 3 Department of Chemistry, Faculty of Science, Saitama University, Saitama, 338-8570, Japan; 4 Department of Medicine V (Hematology, Oncology & Rheumatology), University of Heidelberg, 69120 Heidelberg, Germany; 5 Institute for Integrated Cell-Material Sciences (WPI iCeMS), Kyoto University, 606-8501, Kyoto, Japan

## Abstract

Using planar lipid membranes with precisely defined concentrations of specific ligands, we have determined the binding strength between human hematopoietic stem cells (HSC) and the bone marrow niche. The relative significance of HSC adhesion to the surrogate niche models via SDF1α-CXCR4 or N-cadherin axes was quantified by (a) the fraction of adherent cells, (b) the area of tight adhesion, and (c) the critical pressure for cell detachment. We have demonstrated that the binding of HSC to the niche model is a cooperative process, and the adhesion mediated by the CXCR4- SDF1α axis is stronger than that by homophilic N-cadherin binding. The statistical image analysis of stochastic morphological dynamics unraveled that HSC dissipated energy by undergoing oscillatory deformation. The combination of an *in vitro* niche model and novel physical tools has enabled us to quantitatively determine the relative significance of binding mechanisms between normal HSC versus leukemia blasts to the bone marrow niche.

Mounting evidence has indicated that cellular and humoral determinants in the microenvironment play an essential role in governing the balance between self-renewal and differentiation of somatic stem cells. In the case of hematopoietic stem cells (HSC), adhesion to the niche in the bone marrow (BM) has been shown to maintain the dormancy of the most primitive HSC. The cellular determinants that might play a role include mesenchymal stromal cells (MSC), osteoblasts (OB), and vascular endothelial cells^1^,^2^,^3^. These cell types serve as surrogate niche to support HSC maintenance^2^,^4^,^5^. In the murine system, long-term HSC have been reported to adhere to N-cadherin expressing, spindle-shaped osteoblasts^1^,^6^. It has been demonstrated that human CD34^+^ cells expressing N-cadherin^7^ are involved in early HSC differentiation^8^. In addition, overexpression of N-cadherin in HSC enhances adhesion and inhibits cell division of HSC *in vitro*, suggesting that N-cadherin-mediated adhesion supports long-term maintenance of the HSC pool^9^. Mobilization and homing of HSC have been shown to be regulated by the sympathetic nervous system leading to secretion of stromal cell-derived factor 1α (SDF1α) by endothelial cells, OB and MSC^10^ and its interaction with the receptor CXCR4 expressed on HSC^11^. This multifunctional cytokine serves as chemoattractant for HSC and is suggested as the major player for HSC trafficking between the niche and peripheral blood^12^. Additionally, SDF1α can be found as an immobilized molecule in the extracellular matrix of the endothelium^13^.

Acute myeloid leukemia (AML) is a clonal disorder of hematopoietic stem and progenitor cells that have lost the ability to differentiate into functional granulocytes or monocytes, hence leading to accumulation of undifferentiated leukemia blasts (LB)^14^. Present evidence indicates that the LB are derived from leukemia initiating cells (LIC). The latter are well protected from cell division by adhesion to the marrow niche, hence are resistant to chemotherapy and responsible for recurrence of disease^15^,^16^. Although HSC and LIC both reside in the microenvironment of the BM^17^,^18^, little is known about the precise molecular mechanisms and the differences in the interactions between marrow niche and HSC on the one hand, and LIC on the other. The differential adhesion of HSC versus LIC to the marrow niche might be exploited for the mobilization of LIC before chemotherapy. To quantitatively define the adhesive strength mediated by specific adhesion molecules binding human HSC to the niche, and to discriminate the potential differences as compared to their malignant counterparts, it is essential to design highly controllable *in vitro* model systems that are based on studies on cellular determinants derived from human origin.

In the present study, we have designed a model of surrogate MSC surfaces by the deposition of planar lipid membranes (supported membranes)^19^,^20^ displaying specific ligands, such as N-cadherin and SDF1α (Fig. 1a). In contrast to commonly used assays that rely on counting the number of adherent HSC on a MSC feeder layer^2^, lipid membranes allow for the precise control of the average lateral distance between ligand molecules, e.g. specific proteins, to nanometer (nm) accuracy. In addition to label-free live cell image analysis with reflection interference contrast microscopy (RICM)^21^, we employed a novel assay utilizing intensive pressure waves induced by laser pulses (Fig. 1b) to quantify the adhesion strength of HSC to the *in vitro* model niche^22^. This technique utilizes a "shock wave" (a pressure wave traveling at a speed beyond the sound velocity) that is induced by a picosecond (ps) laser pulse focused near the substrate surface. The reachable force exerted on a cell (>1 mN) by such pressure waves could be more than six orders of magnitude larger than the typical force range achieved with optical traps^23^ or magnetic tweezers^24^. Such pressure waves are strong enough to detach cells from adhesive surfaces in a non-invasive manner. In contrast to alternative approaches such as peeling off a cell using an AFM tip^25^, this novel assay guarantees statistically reliable data points, i.e. 10–20 cells within 20 min. In addition, we analyzed the shape fluctuation of HSC by calculating the autocorrelation maps and corresponding power spectra in Fourier space in order to extract characteristic spatio-temporal patterns from the morphological dynamics of HSC in response to the *in vitro* model niche^26^,^27^. The use of statistical physics methods has enabled us to identify different modes of shape deformation and motion of HSC as well as to assess the energy dissipation by HSC in the presence and absence of SDF1α, which is usually hidden behind stochastic noises.

## Results

### Quantifying the relative significance of SDF1α and N-cadherin

**Figure 2:**
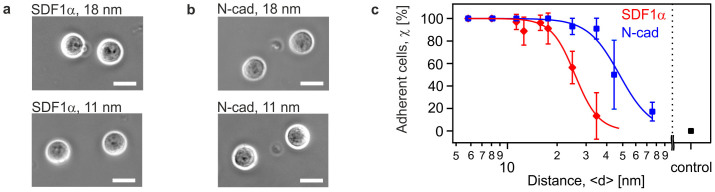
HSC specifically detect changes in intermolecular ligand distance with nm accuracy. Phase contrast images of HSC on membranes displaying (a) SDF1α at <*d*> = 18 nm (top) and <*d*> = 11 nm (bottom) and (b) N-cadherin at <*d*> = 18 nm (top) and <*d*> = 11 nm (bottom) at *t* = 2 h (scale bar: 10 μm). (c) The fraction of adherent HSC on supported membranes, plotted as functions of <*d*_N-Cadherin_> (blue) and <*d*_SDF1α_> (red) could be characterised by an empirical Hill equation (solid lines), identifying a difference in the transition point. Data points represent means ± SD for n = 50 cells.

Figure 2a and 2b represent the phase contrast images of HSC on supported membranes that displayed SDF1α and N-cadherin at <*d*> = 18 nm (top) and 11 nm (bottom), respectively. The fraction of adherent HSC normalized by cells seeded on surrogate niche models was determined by counting the surface densities of adherent cells. As summarized in Fig. 2c (red), an increase in the average lateral distance *<d>* of SDF1α resulted in a significant decrease in the fraction of adherent HSC χ, suggesting a distinct transition from the "bound" state (close to 100%) to "unbound" state (close to the base line). This transition in cell adhesion can be interpreted with the empirical Hill equation^28^:
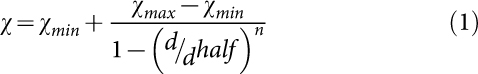
where χ_min_ and χ_max_ stand for the minimum and maximum levels. To exclude any non-specific binding of HSC to the *in vitro* model niche, cells were seeded on a pure phospholipid membrane with no ligand molecules (Fig. 2c, black) as controls. No signs of adhesion was observed on the control surfaces.

**Figure 3:**
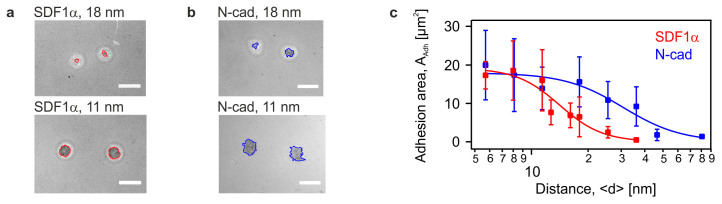
The area of tight adhesion revealed a significant difference between N-cadherin and SDF1α mediated binding. Micro-interferometry images of HSC on membranes displaying (a) SDF1α at <*d*> = 18 nm (top) and <*d*> = 11 nm (bottom) and (b) N-cadherin at <*d*> = 18 nm (top) and <*d*> = 11 nm (bottom) at *t* = 2 h (scale bar: 10 μm). (c) The average area of tight adhesion per cell determined by micro-interferometry, plotted versus <*d*_N-Cadherin_> (blue) and <*d*_SDF1α_> (red) could be characterised by an empirical Hill equation (solid lines) by replacing χ with *A*. Data points represent means ± SD for n = 50 cells.

The unbinding transition could be characterised by the critical distance <*d*_SDF1α_*> ~ 27 nm and the cooperativity coefficient *n* ~ 3, respectively. In contrast, the corresponding results for N-cadherin (blue) exhibited a broader tail to a large intermolecular distance <*d*_N-cadherin_*> ~ 50 nm with a cooperativity coefficient *n* ~ 2, which was consistent with our previous reports on intercellular junctions between HSC and MSC via N-cadherin^7^,^29^. The results suggested that HSC specifically detected slight changes in <*d*> with nm accuracy, and the adhesion mediated by both SDF1α-CXCR4 and homophilic N-cadherin axes could be classified as cooperative reactions. Nevertheless, the determination of the fraction of adherent cells by such an "ensemble counting" does not allow for a clear discrimintation between strongly and weakly adherent cells. In order to gain a deeper insight into the cell adhesion behavior, we have calculated the area of tight adhesion *A_adh_* from individual cells. Figures 3a and 3b represent the Reflection Interference Contrast Microscopy (RICM) images of HSC on supported membranes displaying SDF1α and N-cadherin at <*d*> = 18 nm and 11 nm, respectively. Thus, RICM permitted the quantitative identification of minor changes in *A_Adh_* in relationship to the different average lateral distances *<d>* between ligand molecules. Fig. 3c represents the calculated area of tight adhesion per cell plotted as a function of <*d*_SDF1α_> (red) and <*d*_N-cadherin_> (blue). The area of tight adhesion exhibited a significant decrease from 16 μm^2^ to 7 μm^2^ when the average lateral distance increases from <*d*_SDF1α_> = 11 nm to 18 nm. No adhesion zone could be detected at <*d*_SDF1α_> ≥ 34 nm. The corresponding results on membranes displaying N-cadherin also exhibited a similar tendency, whereas no signs of adhesion could be detected at <*d*_N-cadherin_> > 80 nm. The clear discrimination in the area of tight adhesion at <*d*_SDF1α_> ≤ 18 nm presented in Fig. 3c was in contrast to the ensemble counting results in Fig. 2c, where the cells were merely classified either as "adherent" or "non-adherent". This finding implied that the RICM analysis was much more sensitive for assessment of the difference in cell adhesion. In fact, this might account for the apparent differences in the unbinding transition to a smaller critical distance, <d*> = 14 nm for SDF1α and 31 nm for N-cadherin, obtained by replacing χ to *A* in equation 1. Moreover, the cooperativity coefficients of SDF1α-CXCR4 and N-cadherin axes were *n* = 1.9 and 1.4, respectively, verifying the cooperativity of both interactions. Although an active response of the cell via up- or down-regulation of receptors might affect adhesion on longer time scale, no time-dependent change in the adhesion area *A_adh_* could be detected for HSC on membranes displaying either SDF1α or N-cadherin during a total observation time of 4 h (data not shown).

**Figure 4:**
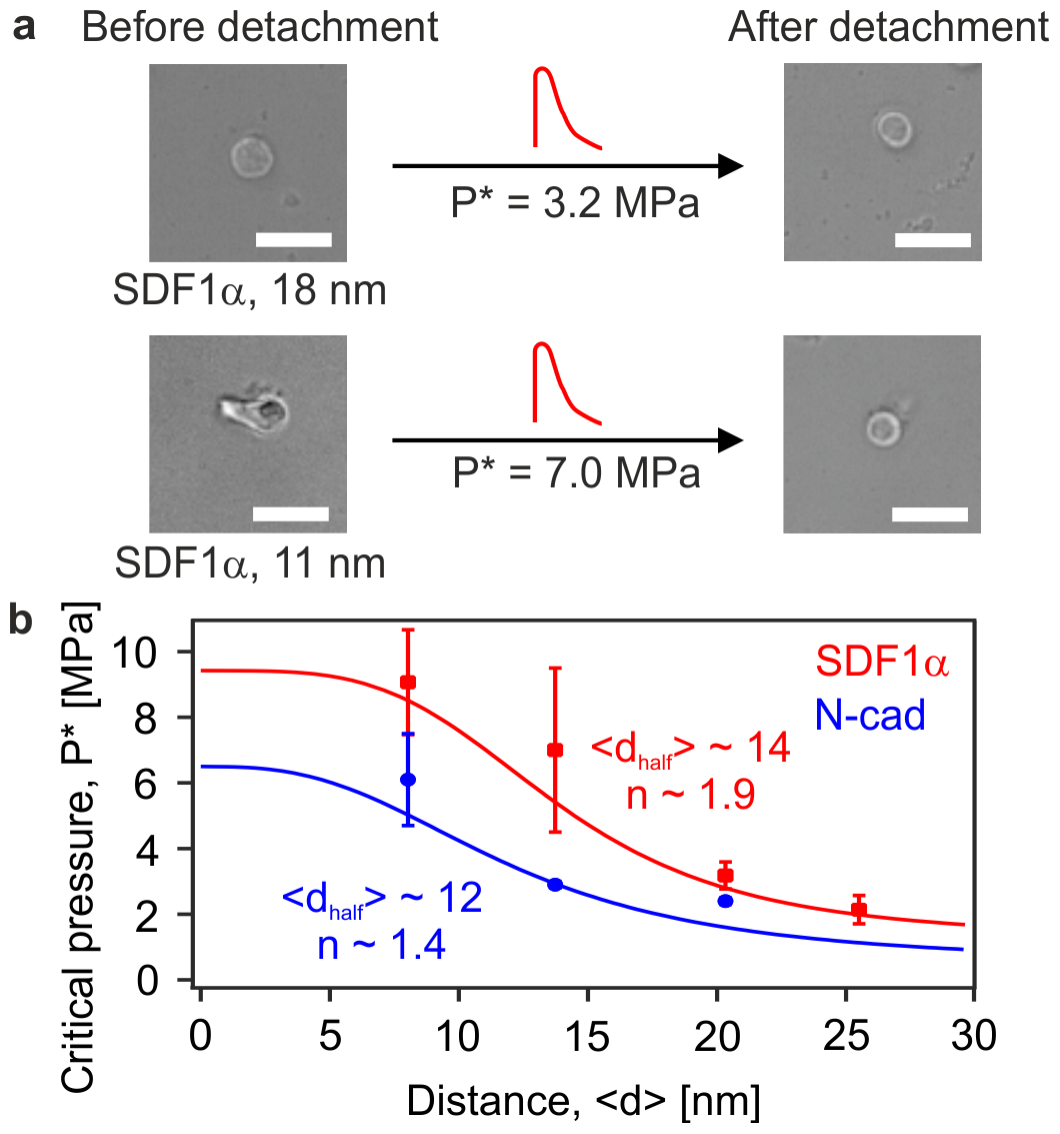
SDF1α mediates a higher adhesion strength compared to N-cadherin. (a) Bright-field images of individual HSC on membranes displaying SDF1α at *<d>* ~ 18 nm (upper) and *<d>* ~ 11 nm (lower) before and after its detachment by pressure waves (red waves) at *t* = 2 h (scale bar: 10 μm). Applying a laser-induced pressure wave, HSC were displaced from the substrate and shifted by about 10 μm. Subsequently, HSC were able to adhere again to the *in vitro* model niche as evidence for its viability. (b) Critical detachment pressures P* determined for HSC on supported membranes at *t* = 2 h, plotted as functions of <*d*_N-cadherin_> (blue) and <*d*_SDF1α_> (red). Data points represent means ± SD for n = 50 cells.

To quantify the mechanical strength of the binding between HSC and the *in vitro* niche model mediated by specific receptor/ligand pairs, we have utilized a novel technique based on cell detachment by pressure waves induced by ultra-short laser pulses^22^. In Figure 4a, single HSC are shown before and after their successful detachment demonstrating that cells were viable and adhered again to the *in vitro* niche model. Figure 4b represents the critical pressures P* for the detachment of HSC plotted as a function of <*d*_N-cadherin_> and <*d*_SDF1α_>. The measurements were performed within the range of <*d*> = 5.5–25 nm, at which the majority of cells were identified as "adherent" (Fig. 2c). The critical pressure P* showed a clear decrease corresponding to the increase in <*d*> within this range, sharing common features with changes in the area of tight adhesion (Fig. 3c). It should be noted that these mechanical assays magnified differences in the strength of HSC binding on the niche model displaying SDF1α and N-cadherin at <*d*>, which could not be identified from the area of tight adhesion (Fig. 3c). For example, the critical pressure P* to detach HSC from the niche model functionalized with SDF1α at <*d*> = 11 nm (P* = 6.4 MPa) was more than two times higher than the corresponding value on membranes displaying N-cadherin at the same average lateral distance (2.8 MPa). Note that the direct comparison between the P* values obtained here and those obtained by other experiments (e.g. AFM, micropipette aspiration) is not possible due to the fact that the characteristic time window for the bond rupture is different by several orders of magnitude. As demonstrated by Merkel *et al*.^30^, the rupture force for individual bonds is strongly dependent on the loading rate. In our experiments, the cells are exposed to the pressure wave only for ~ 80 ns, which is more than 10^7^ times shorter than the characteristic time windows for the other assays^25^,^31^,^32^. In fact, such a short exposure to pressure waves eliminates both inelastic deformation of cells and remodeling of cytoskeleton during the cell detachment from our assays. It is also known that the ligand-receptor binding leads to force generation by the adherent cell towards the substrate. However, such mechanotransduction does not play a role in the case of SDF1α-CXCR4 binding, since SDF1α binds to the extracellular domain of the receptor CXCR4 whose intracellular domain is associated with heterotrimeric G proteins^33^ and thus this process does not involve any direct participation of the cytoskeleton. In the case of homophilic N-cadherin binding, it has been reported that the cell adhesion on substrates displaying immobilized N-cadherin leads to the formation of adherens junctions^34^. Although the extracellular domains of N-cadherin anchored on supported membranes via lipid anchors can undergo lateral diffusion on membrane surfaces, we observed no distinct change in the shape or area of the tight adhesion within our experimental time window (up to 4 h after seeding).

### Influence of soluble SDF1α on adhesion

Since SDF1α is not only expressed by MSC but also released in the extracellular space of the BM microenvironment^10^, the fraction of adherent HSC, the area of tight adhesion, and the critical pressure for cell detachment pressure were measured in the presence or absence (as control) of 5 ng/mL SDF1α, which corresponded to the physiological level of SDF1α in human BM. We have identified a decrease in the adhesion strength by a distinct decrease in the adhesion area and the critical pressure for cell detachment at *t* = 2 h in the presence of soluble SDF1α (Table 1). For example, at <*d*_SDF1α_> = 11 nm, the area of tight adhesion and the critical detachment pressure were reduced almost by a factor of two in the presence of soluble SDF1α. Our finding suggested that the presence of soluble SDF1α was able to shift the dynamic equilibrium between bound and unbound states of CXCR4-SDF1α^35^. The decrease in the HSC adhesion area was much more pronounced compared to that observed in previous experiments on substrates coated with fibronectin, where no significant difference in cell adhesion could be observed until soluble SDF1α was applied at concentrations^36^,^37^. The SDF1α concentrations used in these studies were 1–2 orders of magnitude higher than the physiological concentration we used in this study (5 ng/mL). However, as these studies investigated MSC-HSC interactions and thus cannot disriminate different ligand-receptor pairs, the direct comparison with our results is practically invalid. To define whether soluble SDF1α affects the HSC adhesion mediated by the homophilic N-cadherin axis, we also examined HSC on supported membranes functionalized with N-cadherin in the presence and absence of soluble SDF1α (5 ng/mL) at *<d_N-cad_>* ~ 18–47 nm. The results clearly showed that soluble SDF1α did not affect the fraction of adherent HSC, the average area of tight adhesion, nor on the critical detachment pressure P* mediated by N-cadherin (see Supplementary Table S1).

### Correlation between morphological dynamics and modes of motion

**Figure 5:**
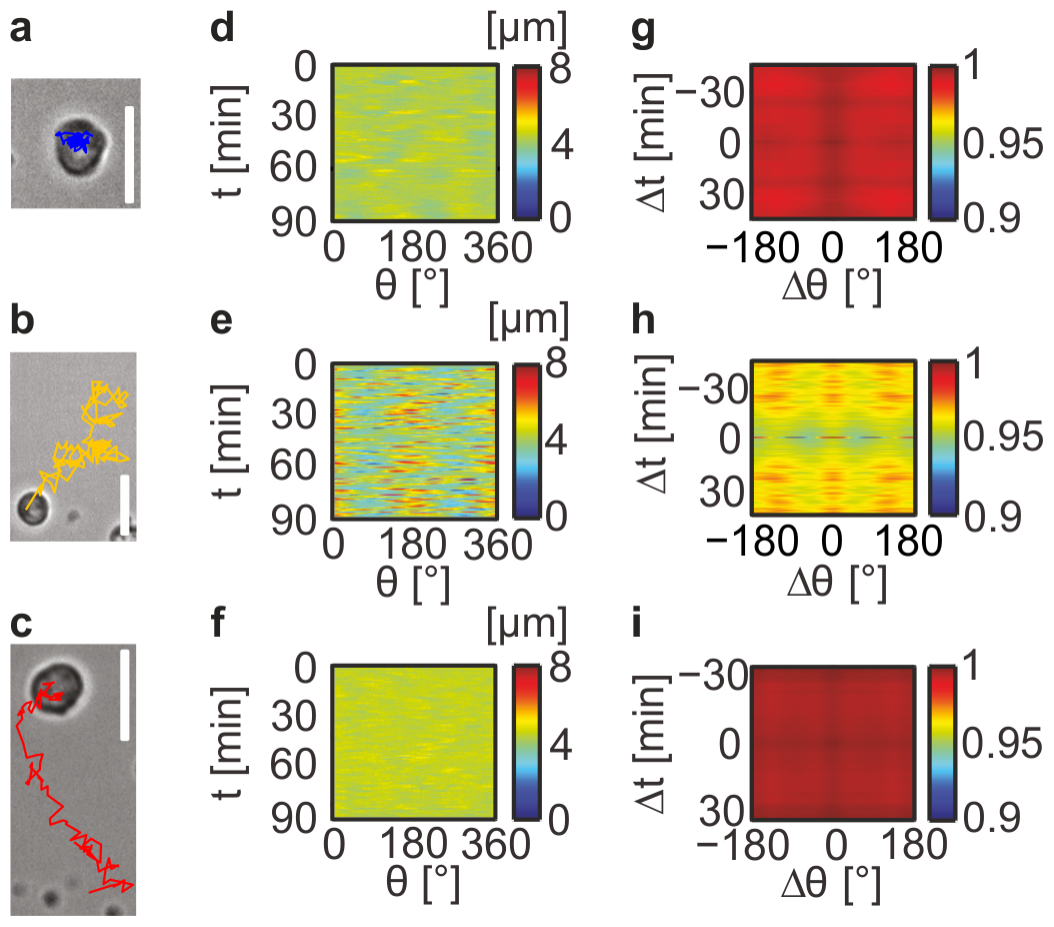
HSC motion can be categorized into three major classes. Morphological dynamics of HSC on supported membranes displaying SDF1α: (a–c) phase contrast images and trajectories over 90 min, (d–f) amplitude *r*(*θ,t*) maps, and (g–i) the corresponding autocorrelation *Γ*_r,r_(*θ,t*) maps. The patterns could be classified into three representative cases as a function of <*d*_SDF1α_>; 6 nm (a,d,g), 18 nm (b,e,h) and 34 nm (c,f,i). (scale bars: 10 μm).

We have then addressed the issue how the HSC-niche interaction is reflected on the morphological dynamics and the mode of locomotion. Such analyses have been applied extensively to characterize the mainly thermal fluctuations in model membranes and erythrocytes^38^,^39^,^40^. In case of nucleated cells the dynamic fluctuations comprise mostly active processes such as the bending and stretching of cellular membranes, rearrangements of molecules embedded therein or the remodeling of cytoskeletal structures^39^,^41^. Thus, the power spectrum analyses yield additional insight into the energy dissipation through cellular deformation. As previously reported, SDF1α is not only a chemoattractant to HSC but also induces cytoskeleton remodeling and thus changes in HSC morphology^37^. To extract characteristic spatio-temporal patterns from stochastic dyamics of HSC, the peripheral edge of the cells was defined from the phase contrast time-lapse images using the contrast in the pixel values (see Supplementary Fig. 2). In the next step, the radial distance *r* between the edge of the cell and the center of mass was plotted as a function of angle *θ* = 0–180° over time *t, r*(*θ*,*t*) yielding the morphological dynamics of the target cell. The characteristic spatio-temporal patterns were extracted by calculating the autocorrelation function *Γ*_rr_(*θ,t*) of the stochastic amplitude map *r*(*θ*,*t*):
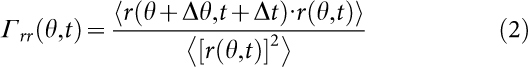


As presented in Fig. 5, using the phase contrast images and the trajectory of HSC tracked over 90 min (a–c), the amplitude maps (d–f) and the autocorrelation maps (g–i), surrogate parameters for cellular locomotion could be subdivided into three major categories: (1) At low average lateral distance of SDF1α, e.g. <*d*_SDF1α_> = 6 nm, the projected HSC morphology remained round and could be characterised by a featureless amplitude map (Fig. 5d). Furthermore, a quick decay of autocorrelation over time (Fig. 5g) suggested that HSC underwent a rotational motion. This finding implied that HSC had little degree of freedom to deform its periphery on sticky surfaces with low average lateral distances *<d>* of adhesive ligands and the translational motion was blocked. (2) At a lateral inter-molecular distance <*d*_SDF1α_> = 11 and 18 nm, HSC extended a pseudopod, followed by an extension in a perpendicular direction (Fig. 5b, e, h). Such an oscillatory deformation could be identified from the periodic appearance of peaks at *θ* = 0/180° and 90/270° in the autocorrelation map (Fig. 5h). This finding suggested that HSC first stretched the pseudopods along the long axis to find the binding spots, then retracted to a round shape, and extended the pseudopods in a perpendicular direction. (3) When the average lateral distance further increased, e.g. <*d*_SDF1α_> = 34 nm, the deformation caused by cell adhesion became less prominent during the translational displacement (Fig. 5f, i).

**Figure 6:**
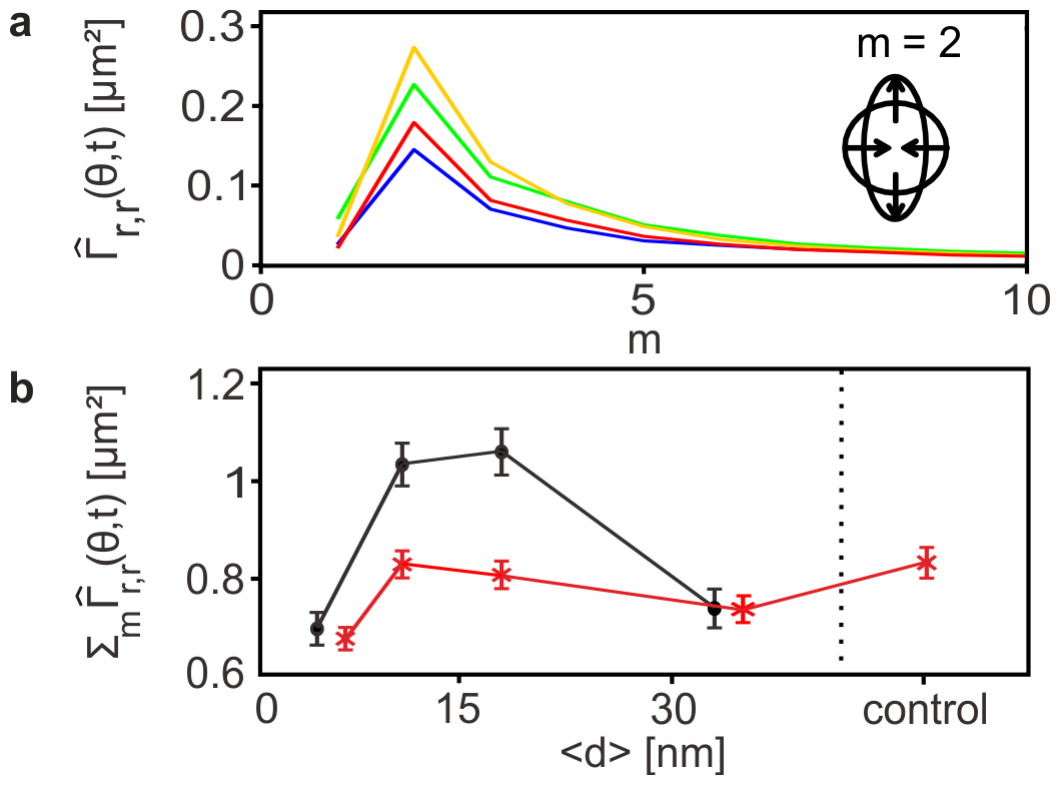
HSC locomotion is mostly based on oscillatory cell deformations. (a) Power spectrum of HSC at various <*d*_SDF1α_> = 6 nm (blue), 11 nm (green), 18 nm (orange) and 34 nm (red), indicating that mode m = 2 (inset) is dominant. (b) Total power in the presence (crosses) and absence (circles) of soluble SDF1α plotted vs. <*d*_SDF1α_>. Data points represent means ± SEM for n = 30 cells of three individual experiments.

Cell deformation, such as bending of cell membranes and remodeling of cytoskeletons^39^,^40^,^41^, is an active processes that consumes energy. Here, we performed the mode analysis of the power spectrum from the Fourier transform of amplitude maps *FT*(*r*(*θ,t*)) for m = 0, 1, 2, 3,… in a similar way to Partin *et al.*^42^ but calculated in the inertial frame with the origin being the center of mass (i.e. the translational mode was not assessed). This enabled one to quantitatively determine the predominant mode of deformation that HSC consumes the energy:
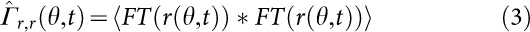


The calculated power spectrum of HSC at various <*d*_SDF1α_> values exhibited peaks at mode number m = 2, implying that the mode of HSC deformation was predominantly oscillatory (inset) independent from <*d*_SDF1α_> (Fig. 6a). In contrast, the deformation at mode m = 1 was suppressed. Moreover, looking at the power spectra calculated for different average lateral distances between SDF1α <*d*_SDF1α_> (indicated by different colors in Fig. 6a), the amount of energy dissipated by the cell through shape deformation did not monotonically follow the changes in the average lateral distance *<d>*, reaching the maximum at around <*d*_SDF1α_> = 11 and 18 nm. This finding suggests that the magnitude of energy consumed by the deformation of HSC is not only determined by the intermolecular distance of ligand molecules, suggesting the presence of a critical intermolecular distance for cells to effectively undergo oscillatory deformations. Fig. 6b represents the calculated total power integrated over all modes as a function of <*d*_SDF1α_> in order to highlight the impact of <*d*_SDF1α_> on the total energy consumed by HSC in the presence (crosses) and absence (circles) of soluble SDF1α. In the absence of soluble SDF1α, the total power exhibited a distinct maximum at around <*d*_SDF1*a*_> = 11 and 18 nm. Such a nonlinear behavior implied that HSC consumes the maximum energy by undergoing oscillatory motion and therefore, they are furthest from equilibrium at <*d*_SDF1α_> = 11 and 18 nm. Remarkably, this range agrees well with the characteristic average intermolecular distances for binding/unbinding transition determined by the pressure wave assay, <*d*_SDF1α_> = 14 nm (Fig. 4). In contrast, the total power exhibited no maximum over the whole range of <*d*_SDF1α_> in the presence of 5 ng/mL soluble SDF1α (Fig. 6b, red), suggesting that consumption of energy by HSC was significantly suppressed in the presence of soluble SDF1α at the physiological level (5 ng/mL). It should be emphasized that the power spectrum analysis in Fourier space only enables one to discriminate the different modes (*m*) of deformation and energy consumption of HSC in the presence and absence of chemo-attractant (SDF1α) at various <*d*_SDF1α_>, which cannot be assessed by the shape analysis in real space.

Similar to cells on membranes displaying SDF1α, we found that HSC on membranes displaying N-cadherin predominantly undergo an oscillatory (mode *m* = 2) deformation (Supplementary Fig. S6). In the absence of soluble SDF1α, the total power 
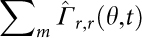
 of HSC on membranes displaying N-cadherin is much less pronounced compared to those on SDF1α-functionalized membranes. The total power 
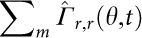
 was hardly influenced by <*d*_N-cadherin_> or by the presence of soluble SDF1α. This finding can be attributed to the different role of N-cadherin from SDF1α. The former serves as the cell adhesion molecule necessary for the anchorage of HSC to the bone marrow niche, while the latter functions as chemo-attractant for HSC migration. Thus, we concluded that the dynamic motion of HSC mediated by N-cadherin is not directly influenced by soluble SDF1α.

### Relevance for clinical stem cell transplantation

**Figure 7:**
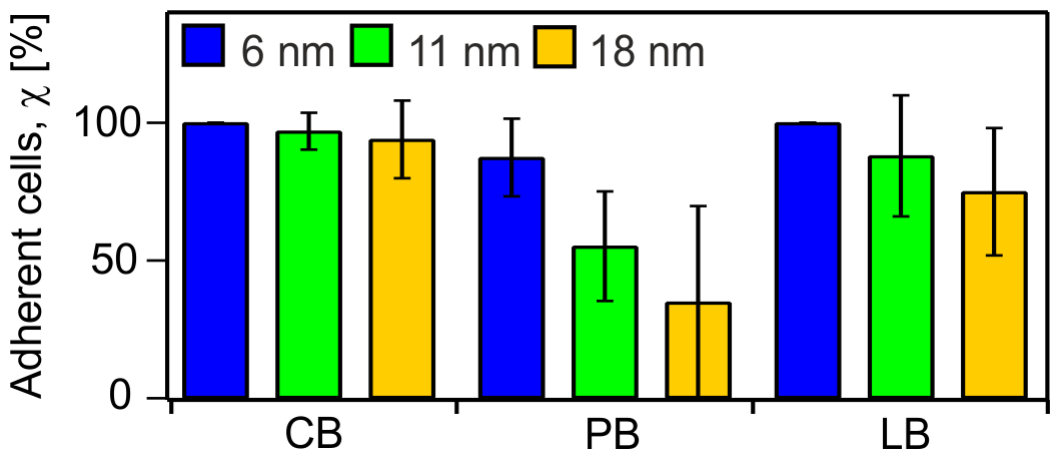
Adhesion dynamics are significantly different between LB, CB and PB HSC. The fraction of adherent cells of HSC from cord blood (CB), HSC from peripheral blood (PB) and leukemia blast cells (LB) at <*d*_SDF1α_> ~ 6 (blue), 11 (green), 18 (yellow), at *t* = 2 h. Bars represent means ± SD for n = 50 cells.

In the case of allogenic stem cell transplantations, HSC from a healthy donor are collected from peripheral blood (PB) after mobilization of HSC from BM with granulocyte colony-stimulating factor (G-CSF)^43^,^44^. It was shown that G-CSF induces the release of neutrophil elastase (NE) and matrix metalloproteinase-9 (MMP-9). This in turn down-regulates levels of SDF1α in BM by enzymatic degradation^45^, follwed by the cleavage of the N-terminus of CXCR4^46^. Thus, the enforced mobilization of HSC led to a decisive distortion of the interaction between SDF1α-CXCR4. We therefore examined the adhesion behavior of HSC from PB to supported membranes functionalized with SDF1α which might be considered as a model of homing of healthy HSC to the BM niche of the patients. Fig. 7 represents the adhesion behavior of HSC from PB as a function of <*d*_SDF1α_>, which was significantly different from that of HSC collected from CB. For example, the fraction of adhered HSC from PB at < *d*_SDF1α_> = 11 nm was 55%, which was much less than that of HSC derived from CB (97%). A similar observation was found for the average area of tight adhesion. This resulted in a shift of the unbinding transition to a critical distance <*d*_SDF1α_*> ~ 10 nm and a cooperativity coefficient *n* ~ 1.3, respectively (Supplementary Fig. S7). The critical pressure for cell detachment was 5.8 MPa at < *d*_SDF1α_> = 11 nm, which was 15–20% less than the corresponding value of HSC derived from CB, 7.0 MPa. We have subsequently examined if the *in vitro* niche model based on SDF1α-functionalized supported membranes could be utilized to identify differential adhesion of CD34^+^ leukemia cells versus normal HSC. In Fig. 7, the adhesion of PB leukemia blasts (LB) from an AML patient was compared with that of PB HSC from a healthy donor. The fraction of adherent LB (88%) was about 1.5 times higher than that of HSC from healthy PB (55%), while the difference from CB HSC (97%) was much less pronounced.

## Discussion

To quantify the adhesive mechanisms between human HSC and the BM niche, we have designed an *in vitro* surrogate surface model of BM niche based on supported membranes displaying specific ligands (N-cadherin and SDF1α) at defined intermolecular distances. This quantitative studies have enabled us to address several open questions in understanding the mechanisms with which the HSC bind to the niche.

First, HSC specifically adhered to the surrogate niche model via homophilic interactions mediated by N-cadherin and heterophilic interactions mediated by SDF1α and CXCR4. This was in stark contrast to the control experiments on pure phospholipid membranes (Fig. 2b), thus excluding the possibility of any non-specific adhesion. Second, the significant correlation between the surface density of adherent HSC and the average intermolecular distance between the corresponding ligand molecules <*d*> demonstrated that HSC sensitively detected minor changes in the intermolecular distance between N-cadherin and SDF1α^7^. Third, the area of tight adhesion calculated from RICM analysis permitted the more precise determination of the characteristic intermolecular distance for binding/unbinding transition (Fig. 3B). Fourth, the laser-induced pressure waves enabled us to quantify the mechanical strength of the HSC adhesion on *in vitro* niche models (Fig. 4b), where the relative significance of two different molecular binding axes was determined in a quantitative manner.

Our results clearly demonstrate that HSC are able to bind to surrogate surfaces displaying N-cadherin. This finding suggests their capability to interact with their niche via the homophilic N-cadherin axis. In fact, this is in good agreement with our previous study demonstrating that N-cadherin is localized at the binding site between HSC and MSC^7^. The direct detection of N-cadherin mediated adhesion of HSC is in stark contrast to other studies which failed to detect any role of N-cadherin^47^,^48^,^49^,^50^. Moreover, we found that the adhesion strength mediated by heterophilic SDF1α-CXCR4 axis was stronger than that mediated by homophilic N-cadherin axis. We have also quantitatively determined the relevance of the counterplay between membrane-bound and soluble SDF1α on the interaction between HSC and the surrogate niche model. As the mobilization/homing mechanism was regulated by a dynamic stimulation of the SDF1α level in the BM, differences in the adhesion strength indicated that the BM niche was able to regulate homing versus mobilization by changing the ratio of soluble versus immobilized SDF1α expressed by MSC.

The statistical analysis of morphological dynamics has demonstrated that HSC predominantly undergo oscillatory locomotion. The power spectra exhibited peaks at around <*d*> = 11 and 18 nm for SDF1α and <*d*> = 34 nm for N-cadherin, respectively, corresponding to the unbinding transition points. This finding is intriguing, as the strongest shape deformation occurred at the binding/unbinding transition and did not scale with the average intermolecular distance between ligand molecules <*d*>. The observed nonlinearity between HSC dynamics and <*d*> implies that HSC was furthest from equilibrium around this <*d*>, dissipating the maximum energy by undergoing oscillatory motion. The total power in the presence of soluble SDF1α (5 ng/mL) was always smaller than the corresponding values in its absence, suggesting that the energy dissipation by HSC is strongly damped in the presence of soluble SDF1α. It should be noted that all the previous *in vitro* studies relying merely on the phenomenological observation needed a concentration of soluble SDF1α which was 1–2 orders of magnitude higher than ours in order to describe the change in cell morphology caused by SDF1α^36^,^37^. This finding demonstrated that the analysis of morphological dynamics of live HSC in Fourier space (power spectrum calculation) is a very powerful tool to extract the non-equilibrium fluctuation dynamics of HSC, which is hidden behind the noise and thus cannot be detected by the conventional image analysis in real space. Our results implied that soluble SDF1α not only interfered with the strength of adhesion mediated by CXCR4-SDF1α but also affected the energy dissipation during the migration of HSC. The combination of quantitative force measurements and defined surrogate surfaces offers a unique advantage over previous *in vitro* adhesion assays on feeder MSC layers^36^,^37^, unraveling not only the relative significance of two key axis mediated by N-cadherin and CXCR4 but also their cross-talk with soluble chemoattractant SDF1α in a quantitative manner. By extrapolating this approach we have compared the adhesion of HSC derived from PB mobilized by G-CSF and that of HSC from CB. The results suggested that G-CSF treatment leads to a lower fraction of adherent cells and a decrease of the adhesion strength as compared to CB HSC. These findings supported the previous studies suggesting that G-CSF induced a release of NE and MMP-9, accompanied by a down-regulation of SDF1α in BM and the cleavage of the N-terminus of CXCR4 by enzymatic degradation^45^ leading to a mobilization of HSC from BM to PB^46^. In contrast, the adhesive strength between LB and the niche might be higher compared to PB HSC. Albeit the number of samples tested was not yet adequate to address this important question, we have demonstrated that the combination of quantitative physical tools will enable us to define the differentiatial adhesion of normal HSC versus LSC to the marrow niche. The combination of precisely controlled *in vitro* niche models and new quantitative tools will provide us with significant information for the concept of mobilization of LSC towards the elimation of LSC for long-term remission.

## Methods

### Lipids, proteins

1-stearoyl-2-oleoyl-*sn*-glycero-3-phosphocholine (SOPC), 1,2-dioleoyl-*sn*-glycero-3-[(N-(5-amino-1-carboxypentyl)iminodiacetic acid)succinyl] (nickel salt) (DOGS-NTA (Ni^2+^)) and 1,2-dioleoyl-*sn*-glycero-phospho-ethanolamine-3-N-(cap biotinyl) (biotin-DOPE) were purchased from Avanti Polar Lipids (Alabaster, USA), and neutravidin from Life Technologies. Recombinant stromal cell-derived factor-1α (SDF1α) with and without biotin tags and human N-cadherin with histidine tag were purchased from Almac Group (Craigavon, UK) and R&D Systems Inc. (Wiesbaden, Germany), respectively. For all cell experiments, Iscove's Modified Dulbecco's Media from Life Technologies was used.

### Preparation of supported membranes

Glass slides were cleaned by a modified RCA protocol^51^. Therefore, substrates were sonicated in acetone, ethanol, methanol and water for 3 min, subsequently. Then, they were immersed in a solution of 1:1:5 (v/v/v) H_2_O_2_(30%)/NH_4_OH(25%)/H_2_O and sonicated at room temperature for 3 min, before soaking them at 60°C for another 30 min. Thereafter, substrates were rinsed intensively with ultrapure water, dried at 70°C and stored in a vacuum chamber at room temperature. Cell incubation chambers were prepared by sealing microscopic grade 256 × 75 mm^2^ glass slides from Gerhard Menzel GmbH (Braunschweig, Germany) to bottomless plastic fluidic channels (μ-Slide VI^0.4^) from Ibidi (Martinsried, Germany) in case of RICM experiments. For picosecond laser pulse detachment assays, round microscopic grade glass slides with a diameter of 28 mm were bonded to bottomless culture dishes from Ibidi. Polydimethylsiloxane produced from base and curing agent (SYLGARD184, Dow Corning Co., USA) served as bonding agent for both chambers.

Stock solutions of lipids in CHCl_3_ (5 mg/mL) were mixed to obtain the desired concentration of either DOGS-NTA (Ni^2+^) or biotin-DOPE in SOPC. After evaporation of CHCl_3_ under a gentle nitrogen stream and storage under vacuum overnight, the lipids were re-suspended in HBS and sonicated with a titanium microtip sonicator S3000 (Misonix Inc., Farmingdale, USA) for 30 min to obtain small unilamellar vesicles (SUVs). To remove any residual titanium particles, vesicle suspensions were centrifuged (Eppendorf, Hamburg, Germany) for 10 min at 13400 g. Thereafter, SUV suspensions (1 mg/mL) were stored at 4°C. Solid-supported membranes were prepared by vesicle fusion^19^. Specifically, SUV suspensions were injected into the sealed cell incubation chamber and incubated for 30 min at 40°C, followed by intensively rinsing with HBS buffer (150 mM NaCl, 10 mM Hepes, pH 7.5) to remove excess SUVs.

### Functionalization of supported membranes N-cadherin and SDF1α

Supported membranes doped with DOGS-NTA (Ni^2+^) were incubated with nickel buffer (1 mM NiCl_2_.6H_2_O, 150 mM NaCl, 10 mM Hepes, pH 7.5) for 45 min to saturate the nickel chelating nitrilotriacetic acid (NTA) headgroups. The buffer was exchanged to calcium buffer (1 mM CaCl_2_.2H_2_O, 150 mM NaCl, 10 mM Hepes, pH 7.5), then human recombinant His6 N-cadherin was added (10 μg/mL) and incubated for 12 h at room temperature. For the immobilization of SDF1α, supported membranes doped with biotin-DOPE were incubated with neutravidin solution (40 μg/mL) for 2 h at room temperature. After removing the neutravidin, biotinylated SDF1α (10 μg/mL) was added. Since the anchor lipids are monomerly incorporated into the matrix lipids, the average lateral distance between lipid anchors *<d>* and thus proteins can be estimated from the molar fraction x of lipid anchors by inserting the value of the lipid area of *A*_lipid_ ~ 65 Å^2^ 52,53.
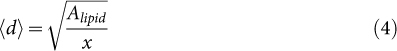


For both N-cadherin and SDF1α, excess proteins were removed by rinsing extensively with cell culture medium prior to the cell adhesion experiments, and the samples were equilibrated at 37°C. As we previously reported, the binding of his-tagged cadherins to NTA lipids and the neutravidin-biotin binding were confirmed using quartz-crystal microbalance with dissipation^52^ and grazing incidence X-ray fluorescence^54^,^55^.

### Isolation of human HSC

All of the experiments involving the use of human HSC were approved by the Ethics Committee of the Medical Faculty, University of Heidelberg, Germany and performed after obtaining informed consent from all voluntary donors in accordance with relevant guidelines and regulations. Human HSC, defined in this study as CD34^+^ cells, were derived from umbilical cord blood (CB) or from healthy allogeneic stem cell donors. The latter had received a mobilization regimen with G-CSF (10 μg/kg bw per day subcutaneously for 5 days) and a sample of 60 ml of peripheral blood (PB) was taken for this study prior to leukapheresis. HSC were isolated as previously described^7^,^56^. Briefly, mononuclear cells (MNCs) were isolated by density gradient centrifugation using the Ficoll–Hypaque technique (Merck KGaA, Darmstadt, Germany). CD34^+^ cells from the MNC fraction were enriched by labelling with magnetic microbeads and sorted twice using an affinity column with the AutoMACS system (all Miltenyi Biotec GmbH, Bergisch-Gladbach, Germany). The cells were allowed to rest for at least 2 hours at 37°C and 5% CO_2_ before further use and were stored in long-term bone marrow culture (LTBMC) medium, a basal HPC culture medium described by Dexter et al.^57^ consisting of 75% Iscove's modified Dulbecco's medium (IMDM; Life Technologies Inc., Carlsbad, USA), 12.5% FCS, 12.5% horse serum (both Stemcell Technologies Inc., Vancouver, Canada), 2 mM L-glutamine, 100 U/ml penicillin/streptomycin (Life Technologies) and 0.05% hydrocortisone 100 (Sigma–Aldrich Co., St. Louis, USA). Staining with propidium iodide was performed to exclude non-viable cells. Reanalysis of the isolated cells by flow cytometry revealed a purity of > 95% CD34^+^ cells.

### Isolation of leukemia blasts

All of the experiments involving the use of CD34^+^ leukemia blasts (LB) were approved by the Ethics Committee of the Medical Faculty, University of Heidelberg, Germany and performed after obtaining informed consent from the voluntary donor in accordance with relevant guidelines and regulations. LB were collected from a patient with newly diagnosed acute myeloid leukemia (AML). LB from PB were isolated as described above for HSC from CB. After the isolation, the cells were stored in liquid nitrogen at 77 K.

### Cell adhesion experiments

HSC cell sample in LTBMC was separated, one portion was supplemented with soluble SDF1α (5 ng/mL) and both samples were pre-incubated for 2 h at 37°C and 5% CO_2_. Before cell adhesion experiments, culture medium was exchanged to pre-warmed IMDM and cells were seeded at a density of 1 × 10^5^ cells/cm^2^ into cell incubation chambers in the absence and presence of soluble SDF1α (5 ng/mL). The viability of HSC was assessed before seeding and after 2 h and 4 h of seeding HSC on the *in vitro* niche model by performing a dye exclusion test using the diazo dye trypan blue. Additionally, cells were stained using Annexin V for apoptosis. In both cases and for all time points a cell viability of ~90% was obtained, which did not change during the time period of the experiments (see Supplementary Fig. S8 and S9).

### Reflection interference contrast microscopy

Non-invasive live cell imaging using reflection interference contrast microscopy (RICM) was performed on an Axiovert 200 inverted microscope (Carl Zeiss AG, Oberkochen, Germany) equipped with a PlanNeofluar 63x/1.25 Antiflex oil-immersion objective with a built-in lambda-quarter plate, a filter cube with crossed polarizers and a cell incubation chamber from Ibidi. To observe interference, light emerging from a metal halogenide lamp (X-Cite 120 PC, Lumen Dynamics Group Inc., Ontario, Canada) was filtered using a green filter (λ_ex_ = 546 nm) and passed perpendicular oriented polarizers before and after the first and second transmission across the lambda-quarter plate, respectively. Sequences of 20 consecutive images were acquired for at least 10 positions per condition using an Orca ER CCD camera (Hamamatsu Photonics, Hamamatsu, Japan) with an exposure time set to 0.1 s after 2 h of cell seeding. The aperture diaphragm was slightly opened (illumination numerical aperture (INA) ~ 0.64) in order to avoid multiple reflections within cells and loss of contrast. Image corrections for the parabolic illumination profile and shot noise were undertaken as described previously^58^. The measured intensity was converted into heights, applying the RICM theory for finite INA and multiple reflecting layers, with refractive indices *n_1_* = 1.525 (glass substrate), *n_2_* = 1.486 (lipid membrane), *n_3_* = 1.335 (IMDM medium) and *n* = 1.37 (cytosol^59^). The area of tight adhesion was calculated from the average height and standard deviation in each pixel.

### Pressure wave assay

A Nd:YAG laser system (λ = 1064 nm, τ_L_ = 60 ps, PY 61C-20, Continuum, Santa Clara, CA, USA) was coupled to an inverted microscope (Eclipse TE2000-U, Nikon Europe) equipped with a self-built cell incubation chamber. Pressure waves were induced by focusing picosecond laser pulses over a PlanApo 20 ×/0.75 objective into the sample chamber (1.5 mm away from the region of interests, and 0.1 mm above the surface). To confirm the stability and statistical reliability of the assay, calibration measurements were performed before and after several series of experiments (see Supplementary Fig. 1). To determine the relationship between the pulse energy *E*_pulse_ and the hydrodynamic pressure *P* of the generated wave*,* a piezoelectric hydrophone was placed 1.5 mm away from the focal point of the ps laser pulse and 100 μm above the substrate. Then, a data set was recorded displaying the hydrodynamic pressure *P* as a function of the laser pulse energy *E_pulse_* prior to each cell experiment. Thereafter, the pressure affecting HSC can be deduced from the obtained calibration curve. Supplementary Figure S1 represents the hydrodynamic pressure *P* plotted as a function of *E*_pulse_, yielding *P ∝ (E_pulse_)^0.47^.* The critical pressure P* to detach 50% of adherent cells was defined as a new measure to quantify the binding strength. As the irradiation of an intensive, short laser pulse leads to the generation of a cavitation bubble at the position of the focal point, we carefully chose a measurement position at a distance where potential damage to HSC can be excluded. First, the distance between cells and the focal point of the laser (1.5 mm) was set to a value larger than the maximal possible radius of the cavitation bubble (*R_max_* < 1.4 mm), which is at the same time larger than any damage observed on the *in vitro* niche model (with a maximal radial distance of *R_damage_* < 300 μm). Second, the short separation distance between the focal point of the ps laser pulse and the substrate (~0.1 mm) suppresses any adverse effects due to hydrodynamic liquid jet impacts. Thus, the target cells are not exposed to hot gaseous water^60^.

### Time-lapse imaging

Movies of migrating cells were recorded with a Keyence BZ-9000 (Keyence, Osaka, Japan) microscope, equipped with a Plan Fluor 40x/0.6 air objective. 1–2 positions per channel (and adhesion condition) were chosen and recording of phase contrast images was undertaken over 6 hours with a frame rate of 0,025 Hz. Acquired movies were drift corrected utilizing cross-correlation analysis.

### Statistics

The underlying number of experiments per data point corresponds to three experiments. In summary, the fraction of adhered cells χ, the area of tight adhesion *A_Adh_* and the critical detachment pressure P* were calculated for a total number of 50 cells per data point and data included in the corresponding figures represent means ± standard deviation. Data characterising the morphological dynamics of HSC, comprise a total number of 30 cells per data point and represent means ± standard deviation of the mean.

## Supplementary Material

Supplementary Information Supplementary Information

## Figures and Tables

**Figure 1:**
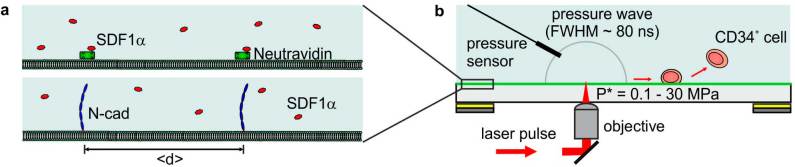
Experimental set-up. Schematic illustrations of (a) the defined surrogate MSC model based on supported membranes and (b) a novel assay to quantify the cell detachment pressure using a laser-induced pressure wave.

**Table 1:** SDF1α shifts the dynamic equilibrium between bound and unbound states of CXCR4-SDF1α. The fraction of adherent HSC *χ*, the average area of adhesion *A*_Adh_, and the critical detachment pressure P* in the presence and absence of 5 ng/mL SDF1α at *t* = 2 h. The experiments were performed at two different average lateral distances of SDF1α, <*d*_SDF1α_> = 11 nm and 18 nm. The values represent means ± SD for n = 50 cells

	χ [%]		A_Adh_ [μm^2^]		P* [MPa]	
<d> [nm]	11	18	11	18	11	18
without SDF1α	97.0 ± 6.7	94.0 ± 14.1	16.0 ± 8.0	6.5 ± 2.0	7.0 ± 2.5	3.2 ± 0.2
with SDF1α	93.1 ± 6.7	52.0 ± 24.4	9.5 ± 6.4	2.0 ± 1.9	3.6 ± 0.7	2.2 ± 0.4
