# Supplementary material for: Quantifying Adhesion Mechanisms and Dynamics of Human Hematopoietic Stem and Progenitor Cells
Source: Sci Rep. 2015 Mar 31;5:9370. doi: 10.1038/srep09370 (PMC5380331; doi:10.1038/srep09370)
Supplement: Supplementary Information [file srep09370-s1.pdf]

Supplementary information

Quantifying Adhesion Mechanisms and Dynamics of Human Hematopoietic Stem and Progenitor Cells

Alexandra S. Burk[1,2], Cornelia Monzel[1], Hiroshi Y. Yoshikawa[1,3], Patrick Wuchter[4], Rainer Saffrich[4], Volker Eckstein[4], Motomu Tanaka[1,2,5]*, and Anthony D. Ho[4]*

[1] Physical Chemistry of Biosystems, Institute of Physical Chemistry, University of Heidelberg, 69120 Heidelberg, Germany

[2] Institute of Toxicology and Genetics, Karlsruhe Institute of Technology, 76021 Karlsruhe, Germany

[3] Department of Chemistry, Faculty of Science, Saitama University, Saitama, 338-8570, Japan

[4] Department of Medicine V, University Hospital, 69120 Heidelberg, Germany

[5] Institute for Integrated Cell-Material Sciences (WPI iCeMS), Kyoto University, 606-8501, Kyoto, Japan

* Corresponding authors: tanaka@uni-heidelberg.de, anthony_dick.ho@urz.uni-heidelberg.de
Phone: (+49) 6221 568000, Fax: (+49) 6221 565813

Calibration curve for pressure wave assay

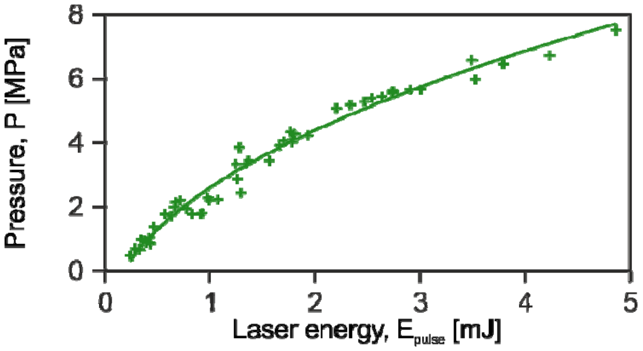

Supplementary Figure S1 Calibration of Pressure Waves. Energy-pressure calibration curve of the pressure wave assay, measured by a piezoelectric hydrophone (Müller-Platte Needle Probe, Dr. Müller Instruments, Oberursel, Germany).

Influence of the laser irradiation on the temperature during the pressure wave assay

The ps laser pulse is focused inside of the cell incubation chamber during the pressure wave assay. At the focal point, heat generation accompanies the formation of a cavitation bubble with a maximum radius of R_{max} < 1.4 mm. Based on the experimental conditions in which the cell detachment assay is performed at a distance of 1.5 mm from the laser focal point, the increase in temperature at this position shall be estimated according to the following two assumptions. First, the laser energy with a maximum value of 10 mJ is fully converted to heat. As the heat transfer to the cell culture medium occurs via multiphoton absorption of which the efficiency is much less than for single photon absorption, this assumption is apparently an overestimation. Second, the medium (specific heat ~ 4.2 J/g·K) is uniformly heated within the radius of 1.5 mm from the focal point. The resultant temperature increase can be calculated according to

$$\Delta T = \frac{1 \times \ 10^{-2}[J]}{4.2 \ \left[\frac{J}{g \cdot K}\right] \times \frac{4\pi \times (1.5\ [mm])^3}{3} \times \frac{1}{2}} = 0.34[\mathrm{K}]$$

Although the underlying assumptions should result in an overestimation of the effect, the calculated temperature increase is negligibly small. Consequently the temperature increase by the laser irradiation does not affect measurements of cell adhesion strength and did not influence the cell viability.

Supplementary Table S1: No influence of soluble SDF1α on the adhesion of HSC mediated by the homophilic interaction of N-cadherin molecules. The fraction of adherent HSC, the average area of adhesion zone, and the critical detachment pressure in the presence and absence of 5 ng/mL SDF1α at t = 2 h. The experiments were performed at two different average lateral distances of N-cadherin, $\langle d_{SDF1\alpha} \rangle$ = 11 nm and 18 nm. Values represent means ± SD for n = 50 cells.

	χ [%]		A_{Adh} [µm^2]		P^* [MPa]	
<d> [nm]	11	18	11	18	11	18
without SDF1α	100.0 ± 0.0	100.0 ± 0.0	13.9 ± 5.5	15.6 ± 6.5	2.9 ± 0.1	2.4 ± 0.1
with SDF1α	100.0 ± 0.0	100.0 ± 0.0	15.0 ± 4.9	13.3 ± 5.0	2.8 ± 0.2	2.5 ± 0.3

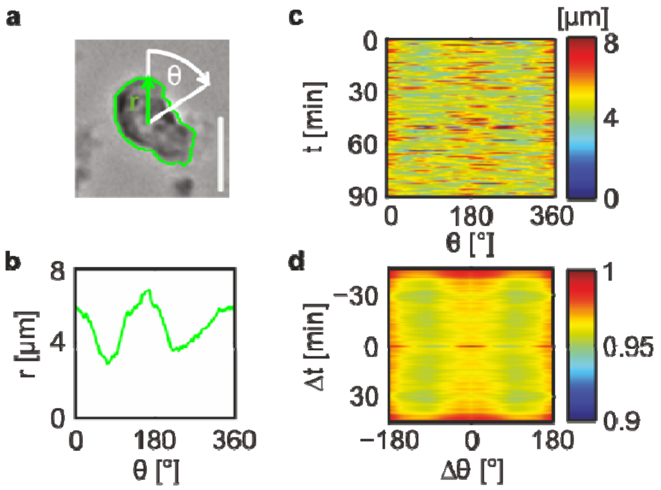

Supplementary Figure S2 Time evolution of cell morphology. (a) Phase contrast image of a HSC on SDF1α functionalized membrane (<d> ~ 11 nm, t = 2 h). The peripheral edge of the cell was determined by the contrast in pixel intensity. Scale bar: 10 μm. (b) The radial distance r plotted in a polar coordinate θ. (c) The amplitude map $r(\theta,t)$ as a function of angle θ over time (t = 60 – 150 min). (d) The autocorrelation $\Gamma_{RR}(\theta,t)$ corresponding to the amplitude map in panel (c).

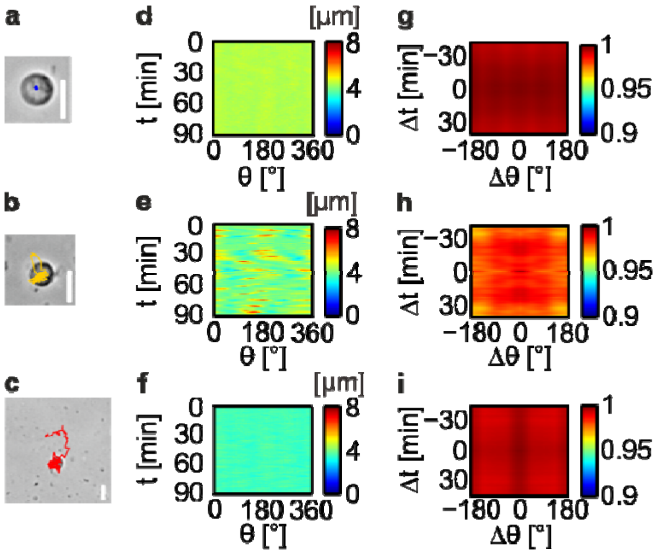

Supplementary Figure S3 Morphological dynamics of HSC on membranes displaying SDF1α in the presence of soluble SDF1α. HSC were observed on membranes displaying SDF1α at <*d*> ~ 6 (blue track), 18 (orange track) and 34 nm (red track) in the presence of soluble SDF1α: (a-c) Phase contrast images and trajectories over 90 min (scale bar: 10 µm), (d-f) amplitude maps $r(\theta,t)$, and (g-i) the corresponding autocorrelation maps $\Gamma_{RR}(\theta,t)$ (t = 60 – 150 min).

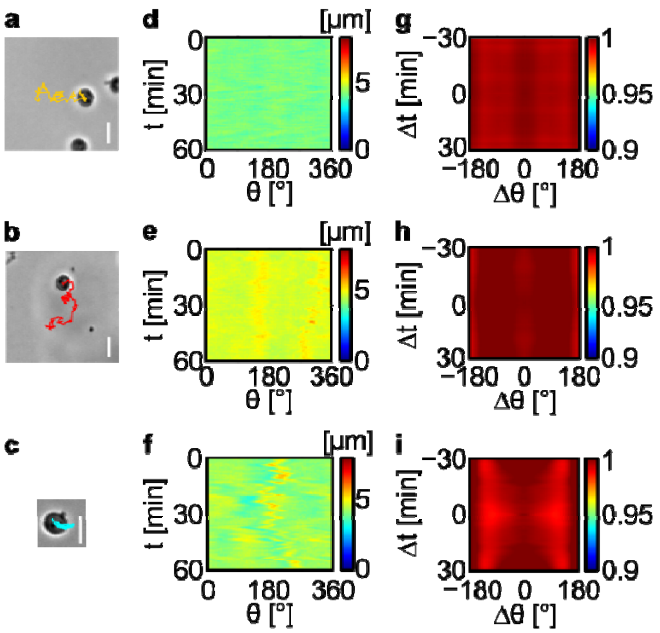

Supplementary Figure S4 Morphological dynamics of HSC on membranes displaying N-cadherin. HSC were observed on membranes displaying N-cadherin at <*d*> ~ 18 (orange track), 34 (red track) and 47 nm (cyan track): (a-c) Phase contrast images and trajectories over 60 min (scale bar: 10 µm), (d-f) amplitude maps $r(\theta,t)$, and (g-i) the corresponding autocorrelation $\Gamma_{RR}(\theta,t)$ maps (t = 60 – 120 min). In contrast to membranes functionalized with SDF1α, HSC on N-cadherin functionalized membranes have smaller trajectories at larger <$d_{N\text{-}cad}$>.

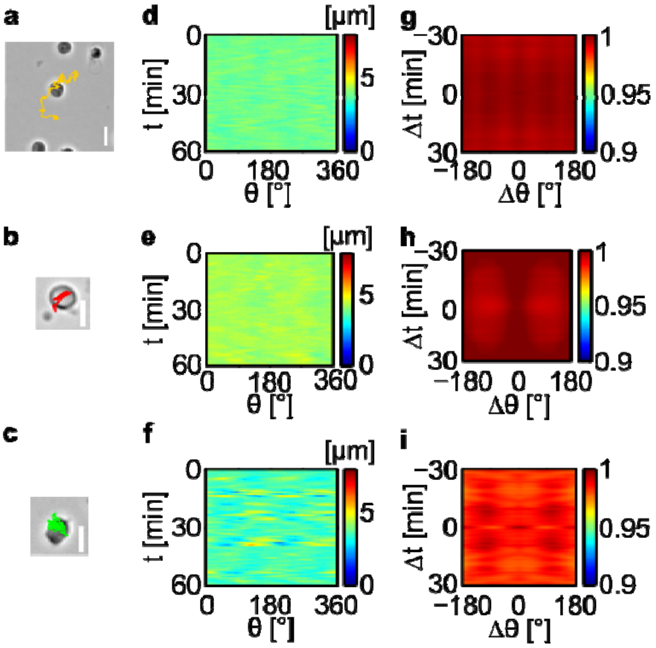

Supplementary Figure S5 Morphological dynamics of HSC on membranes displaying N-cadherin in the presence of soluble of SDF1α. HSC were observed on membranes displaying N-cadherin at <*d*> ~ 18 (yellow track), 34 (red track) and 47 nm (green track) in the presence of soluble SDF1α (5 ng/mL). (a- c) Phase contrast images and trajectories over 60 min (scale bar: 10 µm), (d-f) amplitude maps $r(\theta,t)$, and (g-i) the corresponding autocorrelation maps $\Gamma_{RR}(\theta,t)$ (t = 60 – 120 min).

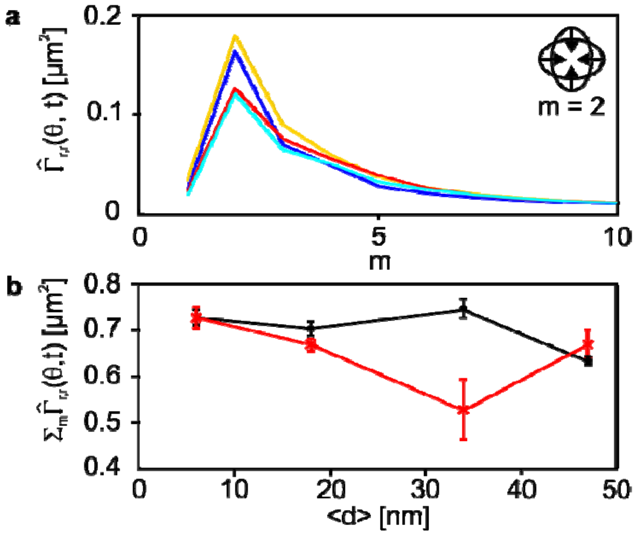

Supplementary Figure S6 Energy dissipation on membranes displaying N-cadherin is not significantly influenced by soluble SDF1α. (a) Power spectrum of HSC at various <$d_{N\text{-}cad}$> = 6 nm (blue), 18 nm (orange), 34 nm (red), 47 nm (cyan) in the absence of soluble SDF1α indicating that mode m = 2 (inset) is dominant at t = 2 h. (b) Total power in the presence (red crosses) and absence (black circles) of soluble SDF1α plotted vs. <$d_{N\text{-}cad}$> at t = 2 h. Data points represent means ± SEM for n = 30 cells.

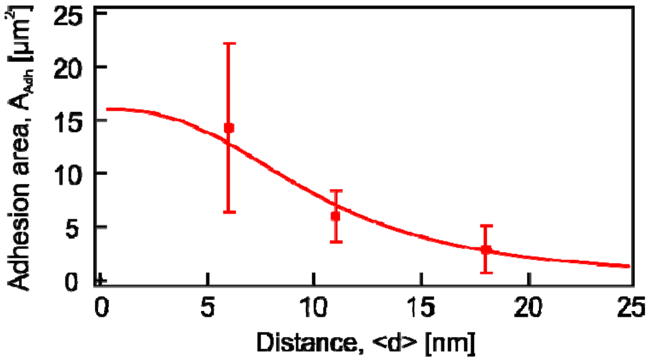

Supplementary Figure S7 Area of tight adhesion of HSC from PB showed a significant shift of the unbinding transition. The average area of tight adhesion per cell determined by micro-interferometry, plotted versus $<d_{SDF1\alpha}>$ (red) could be characterised by an empirical Hill equation (solid line) revealing a critical distance $<d_{SDF1\alpha}{*}>$ ~ 10 nm and a cooperativity coefficient *n* ~ 1.3 for HSC from PB. Data points represent means ± SD for n = 50 cells.

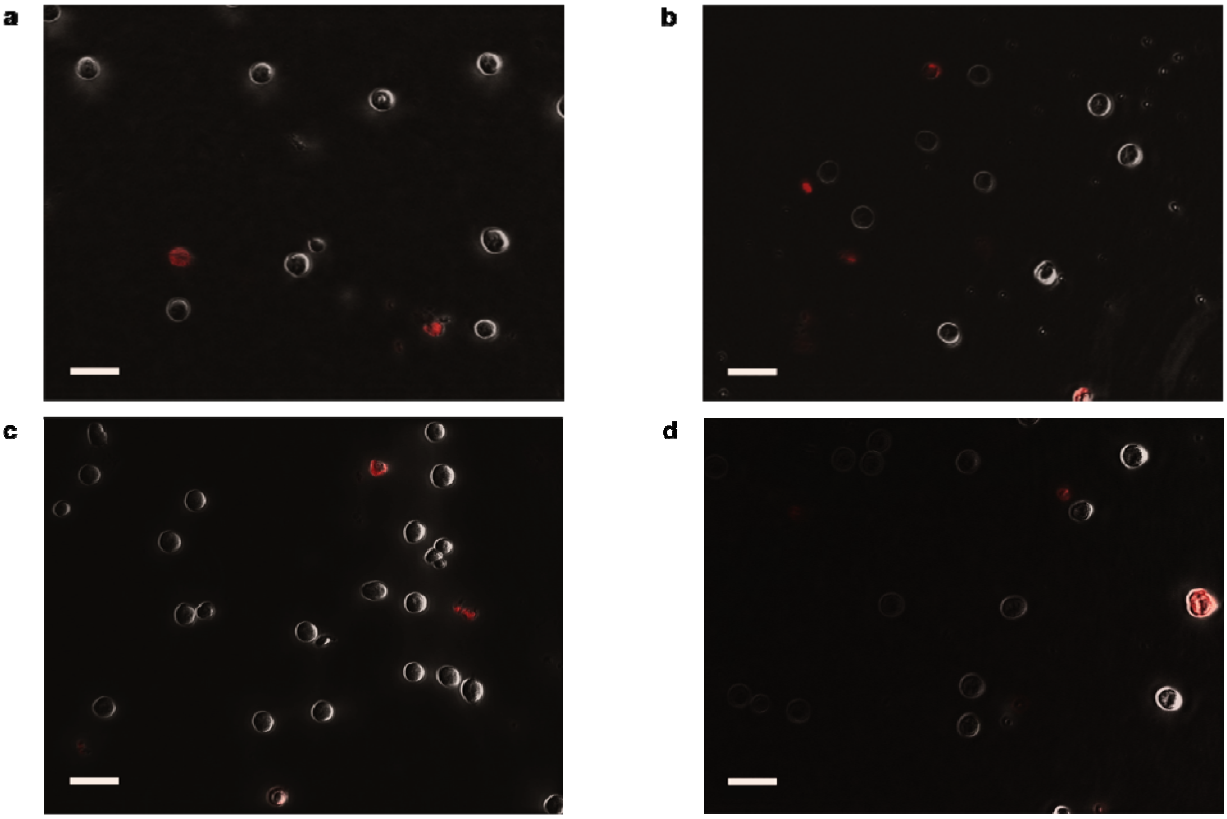

Supplementary Figure S8 Annexin V staining revealed a cell viability of 90 %. Microscopic images represent overlays of phase contrast and fluorescence images of HSC which were seeded on membranes displaying SDF1α (a, b) and N-cadherin (c, d) at an average lateral distance <*d*> ~ 11 nm after 2 (a, c) and 4 h (b, d). Apoptotic cells are shown in red. Scale bars represent 20 μm.

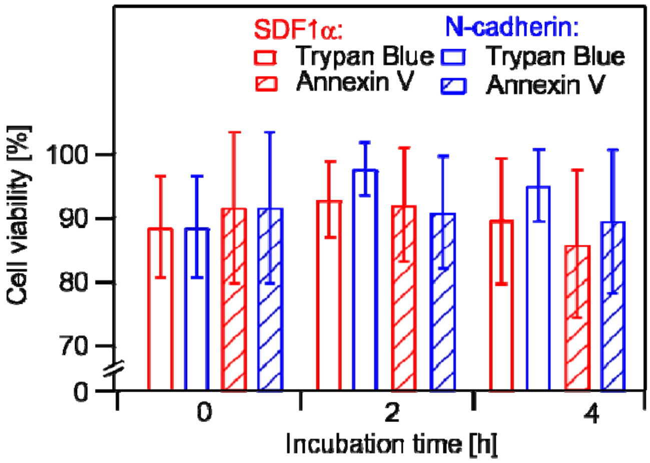

Supplementary Figure S9 HSC viability was observed using a trypan blue and annexin V staining. HSC were seeded on membranes displaying N-cadherin (blue) and SDF1α (red) at an average lateral distance <*d*> ~ 11 nm and cell viability was tested after 0, 2 and 4 h using trypan blue (empty bars) and annexin V (stripped bars) staining. In both cases and for all time points a cell viability of ~ 90 % was obtained, which did not change during the experiment.
